# Cyclooxygenase-2 expression is a prognostic biomarker for non-small cell lung cancer patients treated with adjuvant platinum-based chemotherapy

**DOI:** 10.1186/s12957-014-0426-0

**Published:** 2015-02-06

**Authors:** Katsuhiko Shimizu, Takuro Yukawa, Riki Okita, Shinsuke Saisho, Ai Maeda, Yuji Nojima, Masao Nakata

**Affiliations:** Department of General Thoracic Surgery, Kawasaki Medical School, 577 Matsushima, Kurashiki, Okayama 701-0192 Japan

**Keywords:** Non-small cell lung cancer, Adjuvant chemotherapy, Cyclooxygenase-2

## Abstract

**Background:**

Adjuvant chemotherapy after the resection of stage IB-IIIA non-small cell lung cancer (NSCLC) is now the standard of care based on large-scale phase III trials and a meta-analysis. However, chemotherapy has plateaued in terms of its efficacy, and the search for treatment prediction biomarkers is imperative for the further identification of treatable subgroups. Therefore, we investigated the significance of cyclooxygenase-2 (Cox-2) expression and the applicability of a Cox-2 inhibitor in patients who had received adjuvant chemotherapy.

**Methods:**

We conducted a retrospective review of data from 97 patients who had received adjuvant chemotherapy. The adjuvant chemotherapy consisted of an oral tegafur agent (OT) or platinum-based chemotherapy (PB). The criteria for regimen selection were based on a discussion among the cancer board and enrollment in a clinical trial. Immunohistochemical staining (IHC) for Cox-2 was performed, and the correlation between Cox-2 expression and disease-free survival (DFS) was evaluated.

**Results:**

IHC showed that 56 cases (57.7%) were positive for Cox-2. The rate of Cox-2 expression was similar for the PB and OT groups. Among the patients who received PB, the DFS of the patients with Cox-2 expression was significantly poorer than that of the patients without Cox-2 expression (*P* = 0.017), but there was no significant difference among the patients who received OT (*P* = 0.617). In a multivariate analysis, Cox-2 expression and lymph node metastasis were independent predictors of DFS among patients who received PB.

**Conclusions:**

Cox-2 expression was a powerful predictor of DFS among patients who received PB as an adjuvant chemotherapy. Further study investigating the use of a Cox-2 inhibitor for adjuvant chemotherapy is needed.

## Background

Lung cancer is a leading cause of cancer-related death worldwide. The most effective treatment of early-stage non-small cell lung cancer (NSCLC) is surgical resection. In addition, adjuvant chemotherapy after the resection of stage II-IIIA NSCLC is now the standard of care based on three large-scale phase III trials and an individual patient meta-analysis [1-4]. However, up to 60% of patients with NSCLC with lymph node metastasis relapse after surgery [5,6]. Currently, chemotherapy has plateaued in terms of its efficacy, and the search for treatment prediction biomarkers is imperative for the further identification of treatable subgroups. For unresectable advanced NSCLC, drug selection is usually determined according to histological subtype and gene mutation. The application of these methods to adjuvant treatment is anticipated [7].

Cyclooxygenase (Cox) is the key enzyme required for the conversion of arachidonic acid to prostaglandins (PGs). Two Cox isoforms have been identified and are referred to as constitutive Cox (Cox-1) and inducible Cox (Cox-2); Cox-2 is an inducible enzyme that is activated in response to extracellular stimuli, such as growth factors and proinflammatory cytokines [8]. Some investigators have demonstrated that Cox-2 is constitutively overexpressed in a variety of epithelial malignancies, such as lung, breast, pancreas, colon, esophagus, and head and neck cancers, and Cox-2 overexpression is usually associated with a poor prognosis [9-11]. Recently, a clinical trial performed by Cancer and Leukemia Group B demonstrated that Cox-2 expression was a significant prognostic factor among patients with advanced NSCLC receiving chemotherapy [12].

However, to our knowledge, the prognostic impact of Cox-2 expression has not yet been investigated among NSCLC patients who have received adjuvant chemotherapy. In the present study, we investigated the significance of Cox-2 expression among NSCLC patients who have received adjuvant chemotherapy.

## Methods

### Study population

We conducted this retrospective study in a total of 442 patients with NSCLC who underwent resection at the Kawasaki Medical School Hospital between 2004 and 2010. Of these, 97 patients received adjuvant chemotherapy and were enrolled in this study. None of the patients had received either radiotherapy or chemotherapy prior to surgery. The histological diagnosis of the tumors was based on the criteria of the World Health Organization, and the TNM stage was determined according to the 2009 criteria. Informed consent for the study of excised tissue samples from the surgical specimens was obtained from each patient. This study was conducted with the approval of the institutional Ethics Committee of Kawasaki Medical School (No.1417: Approved on March 11, 2013).

### Adjuvant chemotherapy and follow-up

The adjuvant chemotherapy consisted of an oral tegafur agent (OT) or platinum-based chemotherapy (PB). The criteria for regimen selection were based on a discussion among the hospital cancer board and enrollment in a clinical trial. Basically, OT was selected for patients with stage I (T1bN0M0 and T2N0M0), and PB was selected for patients with stage II and IIIA [4,13]. The OT regimens consisted of tegafur-uracil (UFT) or S1. The PB regimens mainly consisted of carboplatin and paclitaxel, carboplatin and gemcitabine, carboplatin and S1, and other cisplatin regimens. Postoperative radiotherapy was not performed. The schedule for follow-up examinations was arranged on an each individual basis; most of the patients received medical check-ups and chest X-ray films or CT scans at least twice per year. The last follow-up review was performed on June 30, 2014. The median follow-up duration for the determination of disease-free survival (DFS) was 38.6 months (range, 1 to 62 months).

### Immunohistochemical staining

The immunohistochemical analyses were performed using resected, paraffin-embedded lung cancer tissues. After microtome sectioning (4 μm), the slides were processed for staining using an automated immunostainer (Nexes; Ventana, Tucson, AZ, USA). The primary antibodies were used according to the manufacturer’s instructions (Cox-2: Dako Cytomation, (Glostrup, Denmark) CX-294, 1:50 dilution). The slides were scored for the intensity of staining (0 to 3) and the percentages of cells with scores of 0 (0%), 1 (1% to 9%), 2 (10% to 49%), and 3 (50% to 100%) were determined. The immunohistochemistry (IHC) score (0 to 9) was defined as the product of the intensity and the percentage of cells. Cox-2 expression was judged as positive when the IHC score was ≥4 [12].

### Statistical analysis

All the statistical analyses were performed using the SPSS statistical package (version 17.0; SPSS, Chicago, IL, USA). Categorical data were examined using the *χ*^2^ test. The prognostic evaluation was performed based on DFS. DFS was defined as the time until lung cancer recurrence or non-lung cancer death. The impact of Cox-2 expression on DFS was evaluated according to the type of adjuvant chemotherapy (OT or PB). The survival curves were estimated using the Kaplan-Meier method, and differences were evaluated using the log-rank test. Univariate and multivariate analyses were performed using the Cox proportional hazards model. Two-sided *P* values of less than 0.05 were considered statistically significant.

## Results

### Clinical characteristics and chemotherapy regimen

The patients ranged in age from 46 to 80 years (mean, 66.9 years). There were 63 men and 34 women. The pathological stage and histological type at the time of the final pathological examination are shown in Table 1. The most frequent histological type was adenocarcinoma: 65 patients (67.0%) had adenocarcinoma, 17 (17.5%) had squamous cell carcinoma, 8 (8.2%) had large cell carcinoma, and 7 (7.3%) had other subtypes. The patients were classified according to the histopathological stage as follows: 42 patients had stage I, 27 had stage II, and 28 had stage IIIA disease. Of the 97 patients, 51 (52.6%) received PB, and 46 (47.4%) received OT. The most frequent chemotherapy regimen was carboplatin + paclitaxel in the PB group, and UFT in the OT group. Five cases using carboplatin + S1 were included in the PB group.

**Table 1:** **Patient characteristics enrolled in this study (n = 97)**

	**Number**	**%**
Sex		
Male	63	64.9
Female	34	35.1
Age, mean ± SD	66.9 ± 9.0	
Histology		
Adenocarcinoma	65	67.0
Squamous cell carcinoma	17	17.5
Large cell carcinoma	8	8.2
Adenosquamous carcinoma	2	2.1
Pleomorphic carcinoma	5	5.2
Tumor differentiation		
Well	29	29.9
Moderate	29	29.9
Poor	39	40.2
Nodal status		
N0	56	57.7
N1	19	19.6
N2	22	22.7
Pathological stage		
IA	10	10.3
IB	32	33.0
IIA + IIB	27	27.8
IIIA	28	28.9
Chemotherapy regimen		
Platinum-based agent	51	52.6
CBDCA + paclitaxel	35	
CBDCA + gemcitabine	7	
CBDCA + S1	5	
CDDP + others	4	
Oral tegafur agent	46	47.4
UFT	37	
S1	9	

SD: standard deviation; CDDP: cisplatin; CBDCA: carboplatin; UFT: tegafur-uracil.

### Correlations between chemotherapy regimen and clinicopathological characteristics

The PB group had a higher proportion of a pathological lymph node status of N1 or N2 than the OT group (*P* = 0.009), but no significant associations were observed between the chemotherapy regimen and patient age (*P* = 0.248), tumor size (*P* = 0.220), or histological subtype (*P* = 0.897) (Table 2).

**Table 2:** **Patient characteristics enrolled in this study (n = 97)**

**Characteristics**	**Platinum-based Chemotherapy**	**Oral tegaful Chemotherapy**	***P*** **value**
Patients, number	51	46	
Age (mean), year	65.9	68.0	0.248
Sex			0.287
Male	36	27	
Female	15	19	
Histology			0.897
Adenocarcinoma	33	32	
Squamous cell carcinoma	10	7	
Large cell carcinoma	5	3	
Adenoaquamous carcinoma	1	1	
Pleomorphic carcinoma	2	3	
Tumor size (mean), mm	37.7	34.3	0.220
Pathological nodal status			0.009
pN0	22	34	
pN1	14	5	
pN2	15	7	
Cyclooxygenase-2 expression			0.819
negative	21	20	
positive	30	26	

### Cox-2 expression status

An immunohistochemical study showed that 56 cases (57.7%) had a positive Cox-2 expression status. The rate of Cox-2 expression in the PB and OT groups were similar (58.8% vs. 56.5%) (Table 2).

### Prognostic analysis

**Figure 1:**
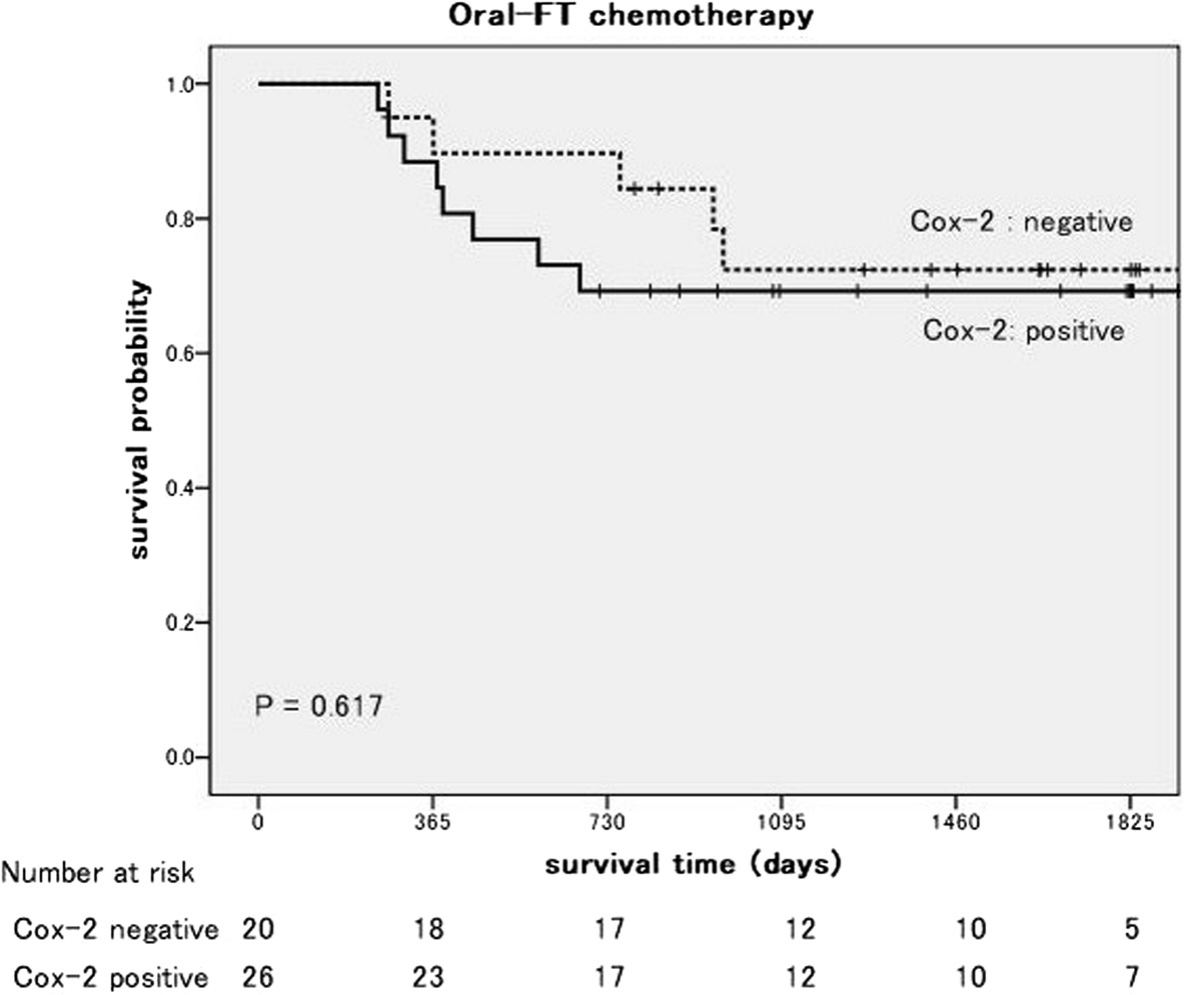
**Kaplan-Meier disease-free survival curves according to cyclooxygenase-2 expression.** Oral tegafur agent: log-rank, *P* = 0.617.

**Figure 2:**
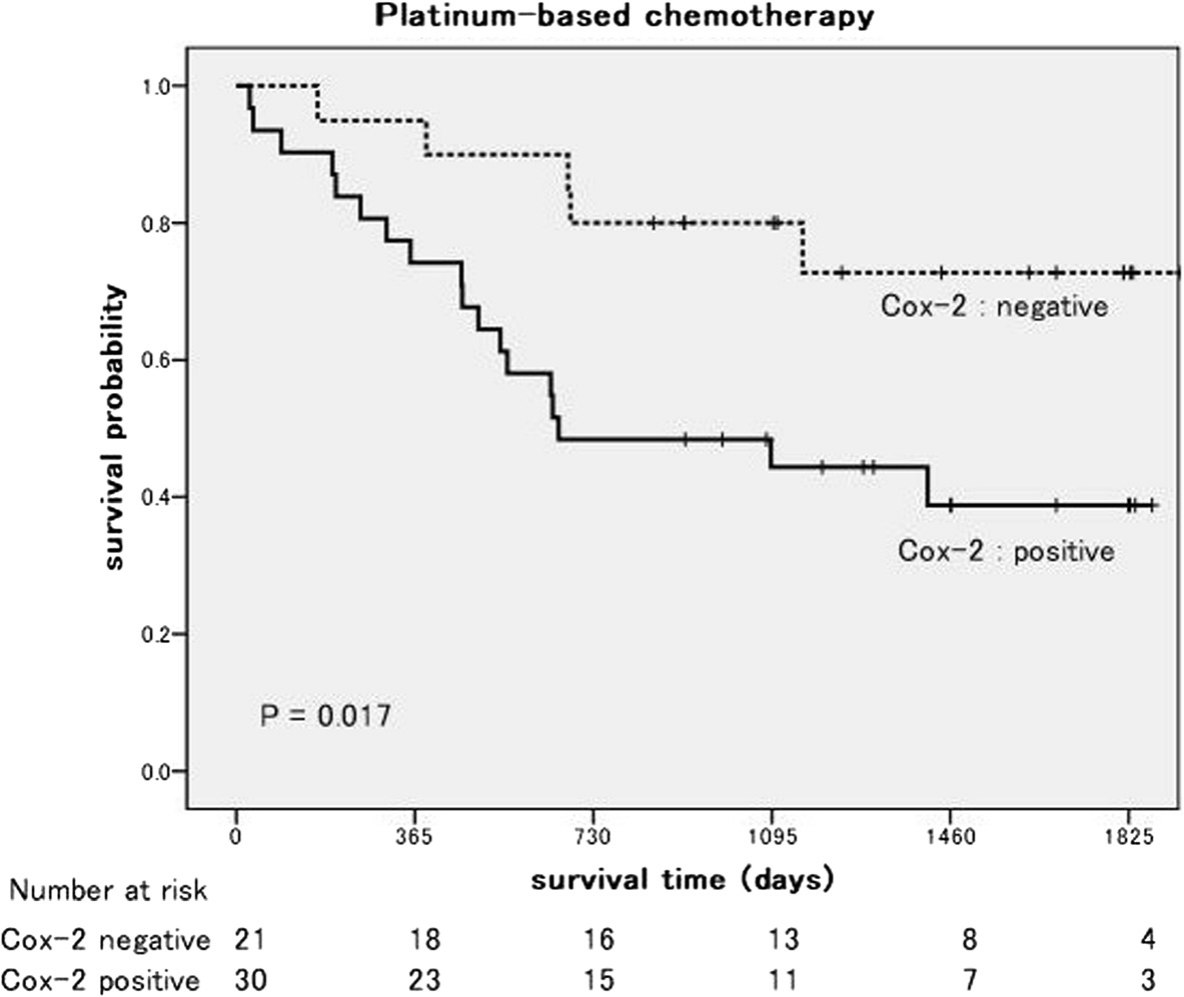
**Kaplan-Meier disease-free survival curves according to cyclooxygenase-2 expression.** Platinum-based chemotherapy: log-rank, *P* = 0.017.

Among the patients who received OT, the DFS of the patients with Cox-2 expression was not poorer than that of the patients without Cox-2 expression (*P* = 0.617, log-rank test; Figure 1). On the other hand, among the patients who received PB, the DFS of the patients with Cox-2 expression was significant poorer than that of the patients without Cox-2 expression (*P* = 0.017; Figure 2). In a univariate analysis, Cox-2 expression and lymph node metastasis were predictors of the DFS. Furthermore, in a multivariate analysis, Cox-2 expression (*P* = 0.011), lymph node metastasis (*P* = 0.030), and vascular invasion (*P* = 0.017) were independent predictors of the DFS (Table 3).

**Table 3:** **Multivariate analysis of factors predicting disease-free survival in adjuvant chemotherapy with platinum-based agent**

	**Univariate**			**Multivariate**		
	**HR**	**95%CI**	***P*** **value**	**HR**	**95%CI**	***P*** **value**
*P* value						
Gender						
Male/female	1.25	0.49-3.18	0.639	2.11	0.77-5.82	0.149
Histology						
non-SQ/SQ	1.89	0.56-6.37	0.303	2.38	0.65-8.74	0.191
Pathological T factor						
T3-4/T1-2	1.81	0.79-4.13	0.160	1.89	0.70-5.11	0.208
Pathological N factor						
Positive/negative	3.63	1.34-9.85	0.011	3.15	1.12-8.85	0.030
Pleural invasion						
p1-3/p0	1.13	0.77-1.67	0.533	0.82	0.50-1.36	0.453
Vascular invasion						
Positive/negative	2.25	0.93-5.52	0.072	3.79	1.26-11.35	0.017
Cox-2 expression						
Positive/negative	3.14	1.16-8.49	0.024	3.96	1.38-11.38	0.011

*P* value was calculated by log-rank test. HR: hazard ratio; 95%CI: 95% confidence interval; SQ: squamous cell carcinoma; Cox-2: cyclooxygenase-2.

## Discussion

Recently, both experimental and clinical studies have revealed that many molecules contribute to the various biological behaviors of malignant tumors including NSCLC. New strategies based on a better understanding of tumor biology are thus needed to maximize the efficacy of current treatments. The associations between these strategies and the response to chemotherapy have been investigated, and the selection of effective chemotherapy regimens based on the evaluation of these biomarkers may improve the clinical outcome of NSCLC patients. PB remains the scaffold upon which combination chemotherapy regimens are assembled for NSCLC patients. As a predictor of the efficacy of PB, the intratumoral expression of excision repair cross-complementing group 1 (ERCC1, a major component of the nucleotide excision repair pathway, is reported to associated with the responsiveness of patients to cisplatin [14,15]. In addition, the intratumoral expression of class III β-tubulin is likely to be associated with the responsiveness to taxanes, such as paclitaxel and docetaxel [16,17]. Moreover, ribonucleotide-diphosphate reductase M1 (RPM1) is likely to be associated with the responsiveness to gemcitabine [18]. On the other hand, OT is widely used in Japan [19,20]. 5-Fluorouracil (5-FU)-derived agents, such as UFT and S1, are effective for patients with NSCLC who have a low expression of thymidylate synthase (TS) [21]. However, evidence recommending the routine clinical use of these agents remains insufficient.

In this study, we investigated the prognostic significance of Cox-2 expression among patients with adjuvant chemotherapy. Several studies have suggested a prognostic and predictive role for Cox-2 expression in NSCLC [22-25]. In a 2006 meta-analysis of the role of Cox-2 in NSCLC, a trend toward Cox-2 overexpression as a prognostic factor affecting the survival of patients with NSCLC was observed, but the heterogeneity among the studies was relatively high [26]. In 2008, Edelman *et al*. reported that Cox-2 expression was a significant prognostic factor in patients with advanced NSCLC (Cancer and Leukemia Group B Trial 30203). Moreover, patients with moderate to high Cox-2 expression had a better tumor response to a Cox-2 inhibitor (celecoxib) in terms of a longer median survival period compared with those not receiving celecoxib [12]. On the other hand, in the NVALT-4 study performed in 2011, Cox-2 expression was not a prognostic biomarker and had no predictive value when celecoxib was added to chemotherapy. However, in a subset analysis, patients with squamous cell carcinoma seemed to perform better when treated with celecoxib [27].

The present study focused on patients who had received adjuvant chemotherapy, and we found that Cox-2 expression was a powerful prognostic factor for patients who had received PB. Regarding postoperative chemotherapy, Cox-2 expression was previously reported not to be a significant prognostic factor when using UFT [28]. The results of this previous report were similar to the results of our study on patients who received UFT or S1. However, whether Cox-2 expression is a significant prognostic factor when postoperative PB is used has been previously reported. The reason for the difference in the effects observed in the PB and OT groups remains unclear. Of interest, the results from a preclinical analysis showed that a Cox-2 inhibitor enhanced the responses to chemotherapy with cisplatin or paclitaxel through the existence of a functional p53-Cox-2 connection in response to DNA damage [29]. From these preclinical findings and the present reported study, the combination of PB, but not OT, and a Cox-2 inhibitor could be promising. Based on these observations and the present study, we propose that a clinical trial examining the use of a Cox-2 inhibitor in platinum-based adjuvant chemotherapy should be conducted.

This study had several limitations that should be considered when interpreting the results. The retrospective study design and the relatively small number of enrolled patients were the major limitations of the present study.

## Conclusions

In conclusion, the present study suggests that Cox-2 expression was a powerful predictor of DFS among patients who received PB as an adjuvant chemotherapy. Further study investigating the use of a Cox-2 inhibitor for adjuvant chemotherapy is needed.

## Abbreviations

5-FU: 5-fluorouracil
Cox: cyclooxygenase
Cox-1: constitutive Cox
Cox-2: inducible Cox
DFS: disease-free survival
ERCC1: excision repair cross-complementing group 1
IHC: immunohistochemistry
NSCLC: non-small cell lung cancer
OT: oral tegafur agent
PB: platinum-based chemotherapy
PGs: prostaglandins
RPM1: ribonucleotide-diphosphate reductase M1
TS: thymidylate synthase
UFT: tegafur-uracil
